# Evidence of transmission of *Clostridium difficile* in asymptomatic patients following admission screening in a tertiary care hospital

**DOI:** 10.1371/journal.pone.0207138

**Published:** 2019-02-11

**Authors:** Prameet M. Sheth, Katya Douchant, Yvonne Uyanwune, Michael Larocque, Arravinth Anantharajah, Emily Borgundvaag, Lorraine Dales, Liz McCreight, Laura McNaught, Christine Moore, Kelsey Ragan, Allison McGeer, George Broukhanski

**Affiliations:** 1 Department of Pathology and Molecular Medicine, Queen’s University, Kingston, Ontario, Canada; 2 Division of Microbiology, Kingston General Hospital, Kingston, Ontario, Canada; 3 Public Health Ontario Laboratories, Toronto, Ontario, Canada; 4 Department of Microbiology, Mt Sinai Hospital, Toronto, Ontario, Canada; Cleveland Clinic, UNITED STATES

## Abstract

**Background:**

*Clostridium difficile* (CD) is the leading cause of infectious health-care associated diarrhea. However, little is known regarding CD carriage and transmission amongst asymptomatic colonizers. We evaluated carriage, characterized strains and examined epidemiologic linkages in asymptomatic colonized CD patients.

**Methods:**

Rectal swabs from asymptomatic patients admitted to the general medicine ward from April 1-June 30 2012 were collected. PCR-confirmed CD colonies were ribotyped and characterized by Modified-Multi Locus Variable Number Tandem Repeat Analysis (MMLVA).

**Results:**

1549-swabs were collected from 474-patients. Overall, 50/474(10.6%) were CD PCR-positive, 24/50 were colonized at admission, while 26/50 were first identified > = 72 hours after admission. Amongst the 50 CD PCR-positive patients, 90% were asymptomatically colonized and 80% of individuals carried toxigenic CD-strains, including ribotype-027 (5/45:11%). MMLVA revealed five-clusters involving 15-patients harboring toxigenic (4/5) and non-toxigenic CD strains (1/5). In two clusters, patients were CD positive on admission while in the other three clusters involving 10 patients, we observed CD transmission from asymptomatically colonized patients to 8 previously CD-negative patients.

**Conclusions:**

We identified increasing rates of colonization during admission to medical wards. MMLVA typing effectively discriminated between strains and suggests that 20% of patients with CD colonization acquired their strain(s) from asymptomatically colonized individuals in hospital.

## Introduction

*Clostridium difficile* (CD) is the leading causes of infectious healthcare-associated diarrhea [1]. CD-disease leads to significant morbidity and mortality including sepsis, pseudomembranous colitis, and toxic megacolon[2, 3]. CD is a gram-positive spore-forming bacillus that is easily transmitted and has been responsible for several health care associated outbreaks in recent years[4]. Asymptomatic colonization of CD is common since the bacteria can colonize the gastrointestinal (GI) tract and make up part of the normal flora. Proper diagnosis of CD infection (CDI) thus requires both the presence of toxin-producing CD and clinical symptoms, such as diarrhea. The development of CD disease is closely related to perturbations of the resident GI flora, which may be due to changes in the host (advancing age), the host’s environment (hospitalization, admission to a long term care facility) or direct alterations to the host GI tract as a consequence of antimicrobial therapy[5–7]. Severity of CD disease is also related to strain characteristics; in particular, the North American Pulso-type 1 (NAP1) or the ribotype-027 strain is associated with more severe disease[8–13].

Although active CD disease poses an obvious organism source in institutional settings, the impact, contribution and transmission of CD from asymptomatic carriers in institutional settings is less clear. Asymptomatic colonization has been observed in 3–5% of healthy adults[14, 15] and up to 50% of patients in hospitals[16, 17] and long-term care facilities[18], representing a large potential reservoir. Several epidemiologic and molecular typing studies have suggested that significant transmission events in institutional settings may be linked to asymptomatic carriers of CD[19–24]. Despite the evidence of the role that asymptomatic carriage plays in the nosocomial transmission of CD in institutional settings, hospitals do not screen patients for asymptomatic CD carriage. This is important due to the potential role asymptomatic carriers could be playing in spore dissemination and additional infections. One study found that detecting carriers and placing them under contact isolation precautions during their hospitalization significantly reduced the amount of health-care associated CD infections in a Canadian hospital[25].

Here we present data on the rates of asymptomatic carriage of CD in patients admitted to a tertiary care hospital in Toronto, Canada. Using both ribotyping and MMLVA in addition to epidemiologic investigations we identified several clusters of asymptomatic CD carriers that harbored both toxigenic and non-toxigenic CD strains and investigated the role that asymptomatic patients play as an underappreciated source for CD.

## Methods and materials

### Study design

A prospective study evaluating the role of asymptomatic carriage of CD in patients hospitalized on one of two 24 bed general medical wards at Mount Sinai Hospital, a tertiary care hospital in Toronto, Canada between April 1^st^ and June 30^th^ 2012. This study was approved by the research ethics board at Mount Sinai Hospital.

### Detection of asymptomatic colonization and infection

Rectal swabs, both e-swabs (Copan Diagnostics, Murrieta, CA) and charcoal swabs (Copan Diagnostics, Murrieta, CA) were collected for all patients as part of admission screening for antimicrobial resistant organisms (ARO) into the general medicine ward. The frequency of repeat swabbing was related to additional screening to monitor a VRE outbreak on one of the general medicine wards. Swabs were assigned unique study codes and stripped of identifiers prior to transfer to the research laboratory.

During the study period CDI was diagnosed clinically and microbiologically confirmed by the detection of the gene for toxin B in stool specimens using the Xpert *C*. *difficile* assay (Cepheid, Sunnyvale CA). Hospital infection control staff identified clinical specimens obtained from study patients for CD testing from February 1 to September 30, 2012 and provided coded information to study staff after all study analyses were complete.

All swabs were alcohol shocked and re-suspended in 1mL of dH_2_O, vortexed, and heat inactivated at 95°C for 10 minutes[26]. The suspensions (10μL) were then planted onto CD moxifloxacin norfloxacin (CDMN) agar (Oxoid Limited, Hampshire, England) and incubated for 48 hours at 35°C under anaerobic conditions (Oxoid Limited, Hampshire, England). Plates were examined after 48 hours. Up to 4 colonies resembling CD were selected, and each re-suspended in one well of a 96-well PCR plate containing 40μL of dH_2_0. The plate was then sealed and DNA extracted using a thermocycler programmed for 10 minutes at 98°C. The PCR plate was centrifuged for 5 minutes at 3000*g* to collect insoluble debris, and 2 μl of supernatant was used in the real-time PCR reaction [27, 28] on the 7900HT Fast Real-Time PCR System (Applied Biosystems, USA). The real-time PCR was a species-specific assay (forward primers; 5’-TTGAGCGATTTACTTCGGTAAAGA-3’ and reverse primers; 5’-CCATCCTGTACTGGCTCACCT-3’) that amplifies a 157bp fragment of a gene of *C*. *difficile* and visualized using a 6-carboxyfluorescein-labelled Taqman probe (TaqMan Probe CGGCGGACGGGTGAGTAACG)[28]. This method detects both toxigenic and non-toxigenic strains of CD [29].

### Molecular typing

PCR-confirmed CD isolates were then ribotyped and further analyzed using Modified-Multi Locus Variable Number Tandem Repeat Analysis (MMLVA)[28]. CD isolates were analyzed by MMLVA and ribotyping as MMLVA has greater discriminatory power. Isolates from the very beginning of the study were not ribotypedas this method was introduced later during the study and has become widely used in CD epidemiology to assist with relatedness identification. Briefly, 2μL of extracted DNA was used and amplified fragments were detected using the 3130xl Genetic Analyzers (Applied Biosystems, USA). For sizing of ribotype and MMLVA fragments, Peak Scanner software v.1.0 was used. Number of repeats for MMLVA was calculated with MS Office Excel and entered to BioNumerics v.7.1 (Applied Maths, Belgium) along with ribotyping data [30]. To calculate relatedness of isolates, a composite data set comprising ribo- and MMLVA typing was used with ribotyping weight of 1X and MMLVA of 2X. As previously described, isolates with a distance of <3% were considered related while identical isolates and those with a difference of ≤ 0.5% were considered to be clustered [27, 28].

Combining MMLVA (with a higher weight of 3X) with ribotyping (with a weight of 1X) ensures against homoplasy seen in some cases when only MMLVA is used. This allows generating results in a single experiment (ribo-MMLVA) when DNA from an isolate is tested in 4 PCR reactions in the same run (MLVA, detection of toxin genes, detection of deletion in tcdC gene and ribotyping). BioNumerics Manhattan distance for clustering was selected over absolute number of tandem repeats for two reasons: (1) widely used assumption that any change in number of repeats per locus should be counted as a difference is not supported by our data on multiple isolates per specimen/patient when minor variation per locus are normal due to a high sensitivity of the method.; (2) significance of increased/decreased number of repeats varies–Cd_A and Cd_C are the most variable, followed by Cd_B and Cd_G and Cd_E being the most stable.

### Epidemiologic investigation

Hospital infection control staff investigated groups of patients with similar MMLVA- and ribotypes retrospectively using patient charts and clinical notes. Information related to time and/or location on the units in the hospital was noted and compared between patients with related strains. Residential postal codes were used to assess the geographical proximity of patients in the community.

## Results

### Patient population and demographics

A total of 1549 rectal swabs were collected from 474 patients (median 2, range 1–20 swabs per patient) admitted to two general medicine wards between April 1^st^ and June 30^th^ 2012 were included in the study (Table 1; S1 Data). The median age of study participants was 74 years (range 17–101 years); the median hospital length of stay was 5 days (range 1–86 days) (S1 Data).

**Table 1:** Participant demographics.

Swabs collected	1549
Patients enrolled	474
Median number of swabs/patients (range)	2 (1–20 swabs)
Median age (range)	74 Years (17–101)
Median length of hospital stay (days)	5 days (1–86 days)
CD positive by culture and PCR (%)	50/474 (10.6%)
Asymptomatic colonization	45/50 (90%)
CD infection	5/50 (10%)
	
CD positive at admission (< 72 hours)	21/45 (47%)
	

### Prevalence of asymptomatic *C*. *difficile* colonization

**Fig 1:**
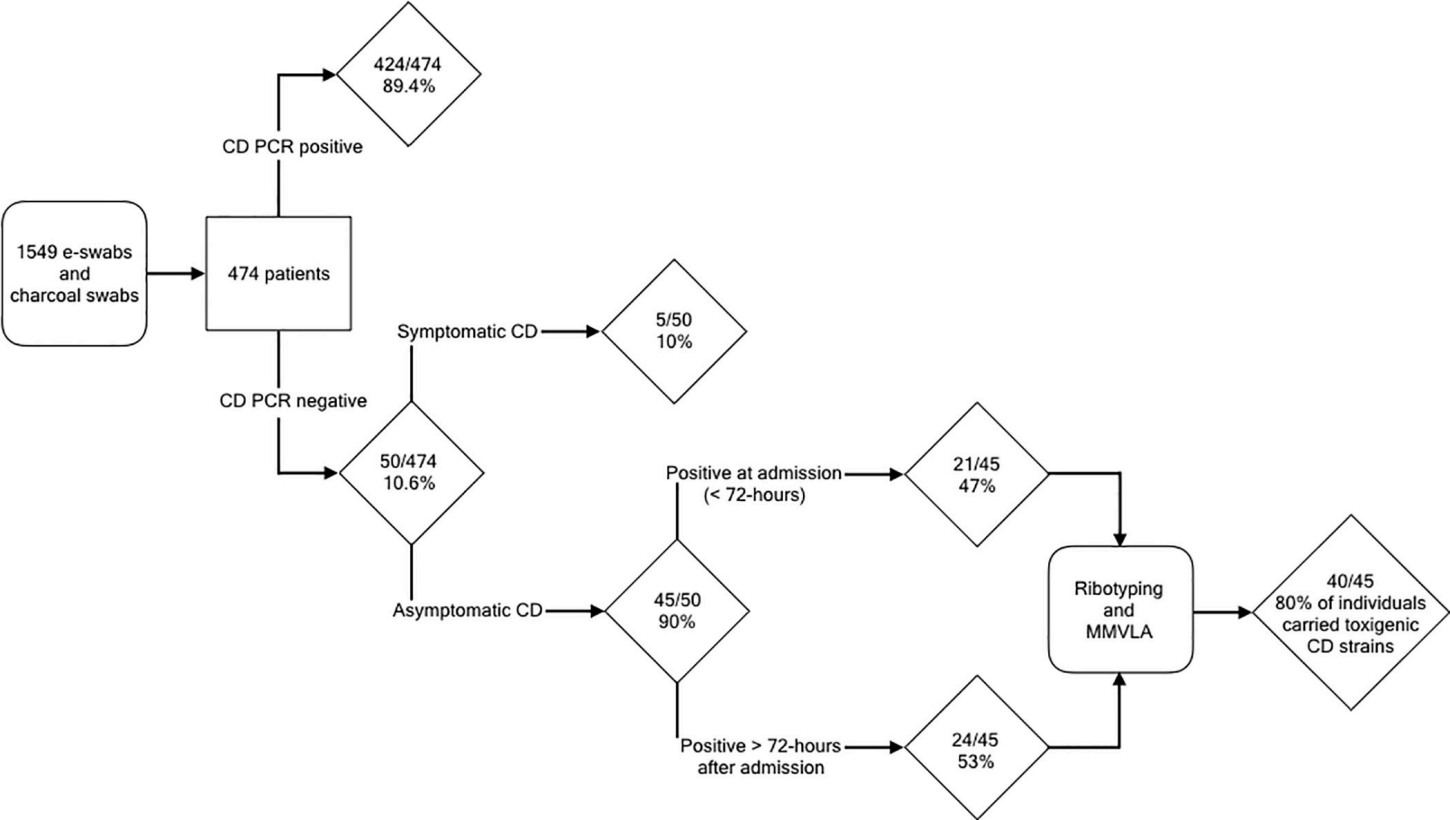
Schematic overview of enrolled study patients.

Overall 50/474 patients (10.6%) (Fig 1) were positive for CD by culture and confirmed by PCR. Amongst participants with positive CD swab(s), the majority (34/50, 68%) had two or more swabs collected. In most cases (30/34; 88%) CD was detected intermittently after the first positive swab and was only consistently detected in 4/34 (12%) patients with multiple swabs collected. In these 4 individuals, CD was detected in all swabs after the first positive swab in a median of three positive swabs collected per patient (range 2–4 positive swabs).

Five of 50 CD positive participants (10% of positive CD or 5/474; 1.1% of the cohort) were clinically diagnosed as having symptomatic CD disease within 3 days of study/hospital admission using a toxigenic-CD specific PCR (Xpert, Cepheid, Sunnyvale, CA) performed on their stool specimens in the clinical microbiology laboratory at Mt Sinai hospital. In addition, 5 patients had negative screening swabs but a diagnosis of CD infection: 3 individuals had negative rectal swabs obtained on the same day as their positive stool specimen; one individual had a negative swab obtained the day after a positive stool specimen (where antibiotic initiation might have reduced CD concentration), and one individual’s only rectal swab was collected 3 days before the stool specimen positive for CD was obtained.

**Fig 2:**
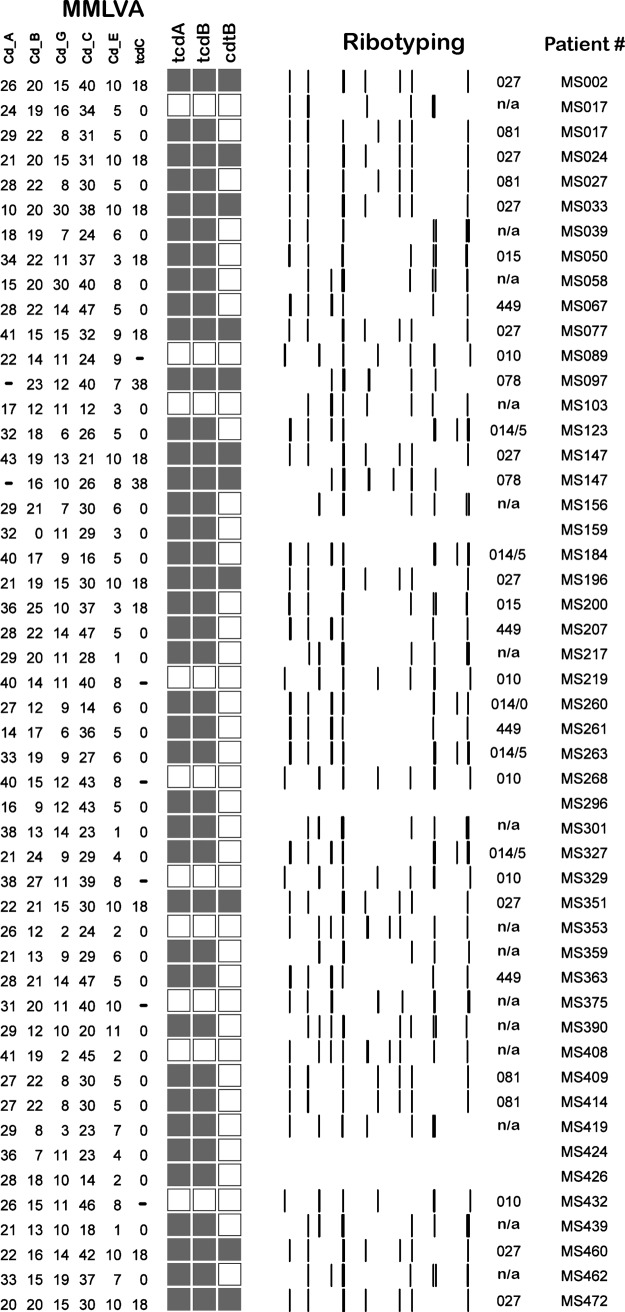
Ribotyping analysis in CD colonized individuals. Asymptomatic carriers were detected carrying several toxigenic (filled boxes) and non-toxigenic (unfilled boxes) ribotypes, including the hyper-virulent ribotype-027 (MS002, MS024, MS033, MS077, MS196, MS351, MS460, and MS472). In some cases an individual was asymptomatically colonized with multiple toxigenic ribotypes including ribotype-027 and ribotype-078 (MS147). Other toxigenic isolates were also prevalent in our patient population including ribotypes 014, 015, 078, 081, 449. Of note, one asymptomatic participant harbored both toxigenic and non-toxigenic CD isolates together (MS017). TcdA–toxin gene A, TcdB–toxin gene B, CdtB–binary toxin gene.

As this study focuses on asymptomatic carriers of CD, the 5 patients with active CD disease on admission were excluded from the study analysis. Data in Fig 2 demonstrate stability of MMLVA profiles in isolates for those patients with multiple specimens tested. Difference of 1–2 repeats is seen only in the most variable loci, Cd_A, Cd_B and Cd_C. The level of MLVA profile variation in multiple isolates per patients sets a baseline of acceptable variability for the strain to be considered the same when clustered isolates come from different patients. Of the 45/474 (9.5%) asymptomatically CD colonized individuals, 21/45 (47%) were colonized at admission (specimen obtained < 72 hours after admission) (Fig 1). The remaining 24/45 (53%) individuals had their first CD positive swab more than 72-hours after admission, with the great majority (23/24; 96%) having had at least one negative swab prior to their first positive swab.

### Molecular characterization of CD isolates

**Fig 3:**
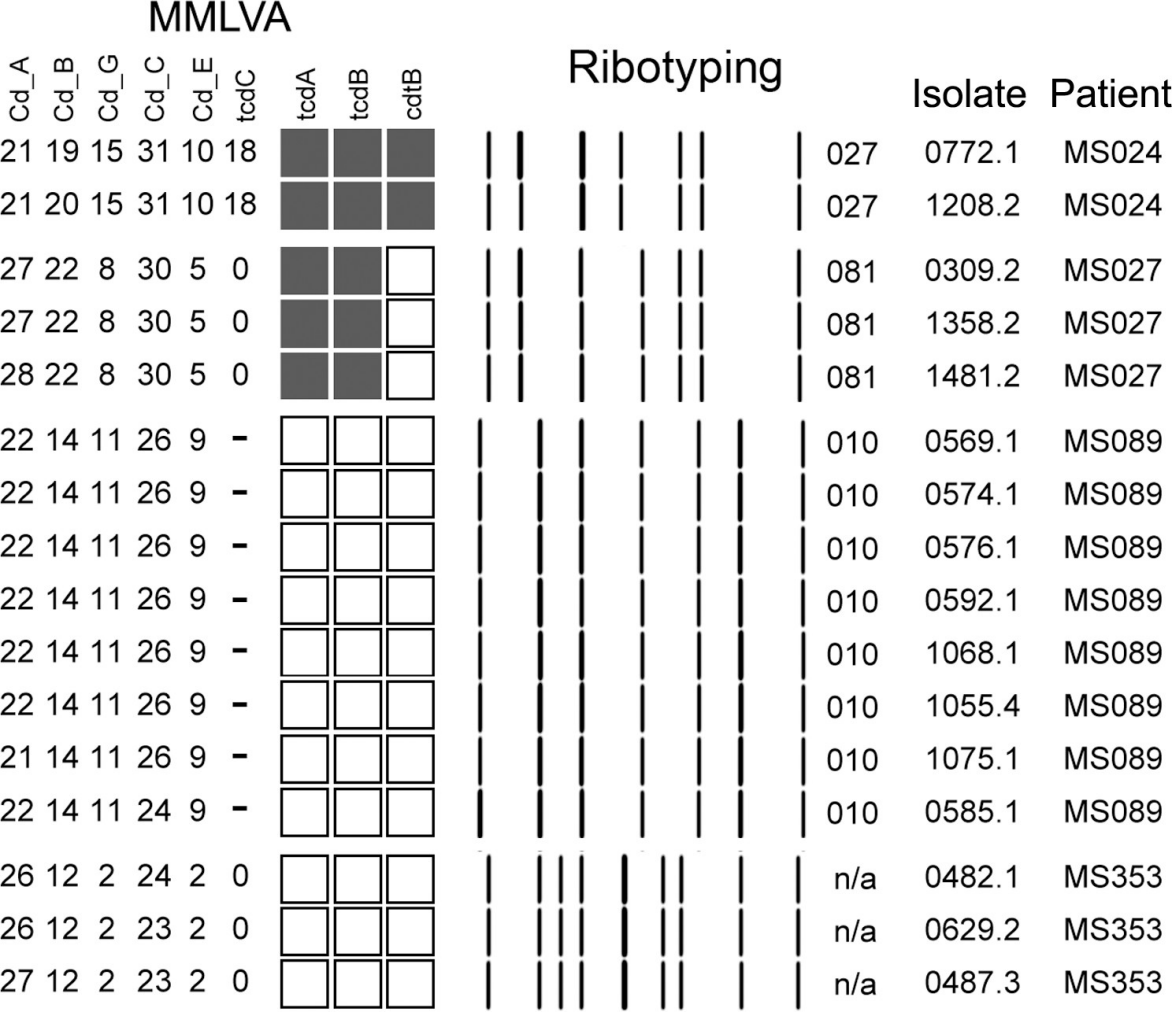
MMLVA profiles from multiple samplings. MMLVA analysis from multiple swabs collected on study participants over several days demonstrated that MMLVA profiling of CD isolates from the same patient over time were consistent and reliable (e.g. MS024, MS027, MS089 and MS353). MMLVA profiles of several specimens from MS024, MS027, MS089 and MS353 collected over time (range; swabs collected 8–67 days apart).

Ribotyping analysis was performed on 72 isolates from 40/45 colonized patients, and revealed that 31/40 (69%) were colonized with non-ribotype-027 toxigenic strains including ribotype-449 (n = 4), ribotype-015 (n = 6), and ribotype-081 (n = 3) (Fig 2).Of those colonized with one strain 9/40 (20%) individuals were colonized with non-toxigenic strains while5/40 (11%) were colonized with the hyper-toxin producing ribotype-027 strain.MMLVA analysis was performed on 85 isolates from 40/45 (90%) asymptomatic carriers. Of the 14 colonized patients with multiple positive specimens collected over 3–23 days, MMLVA profiles were identical in 11 (79%) (Fig 3; pattern in 4 repeated samplings) (S2 Data).

**Fig 4:**
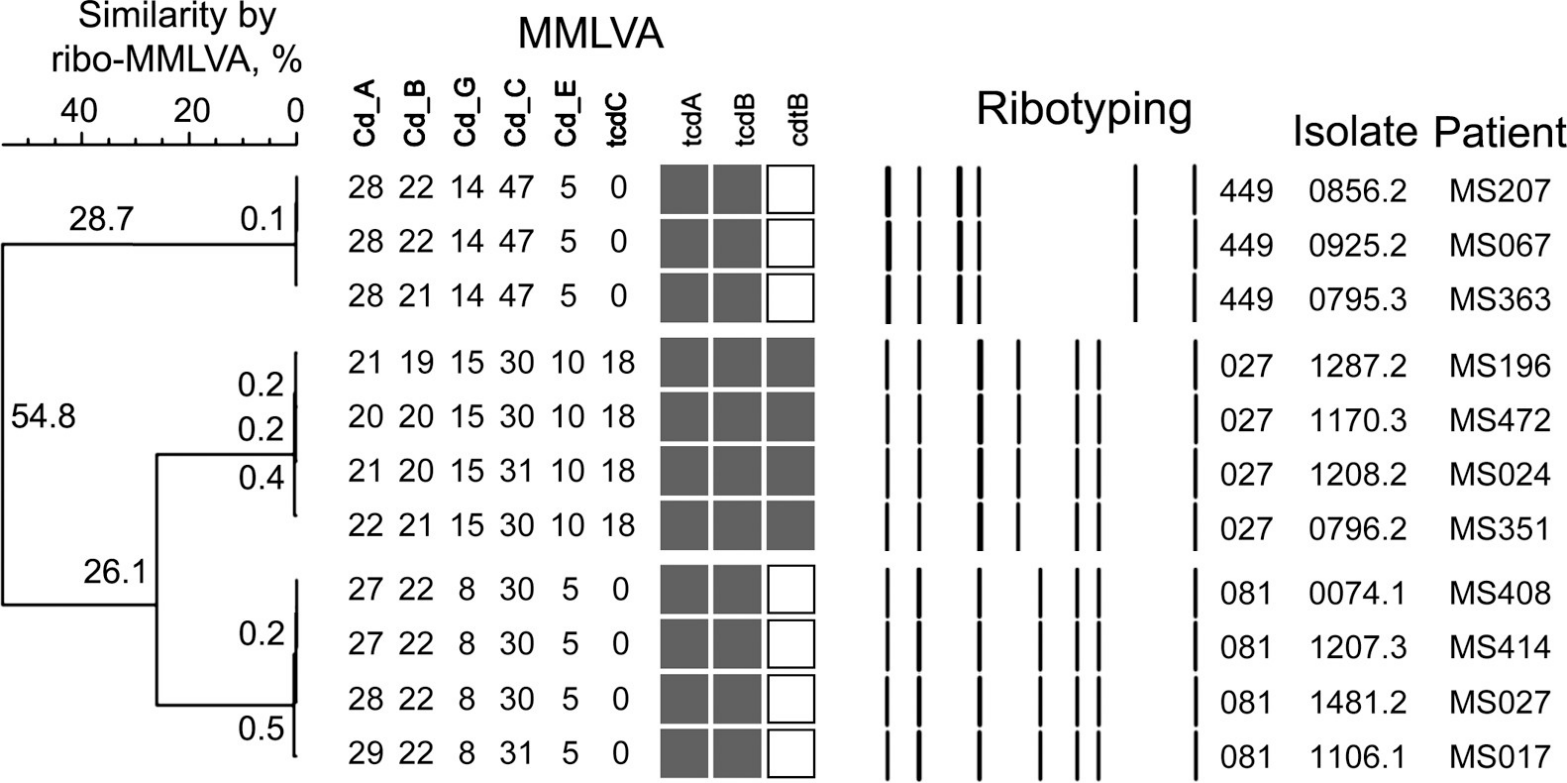
Distance between CD isolates and identifying patient clusters with identical CD isolates. MMLVA identified 5 unique patient clusters that consisted of a total of 15 individuals with highly related CD isolates. The largest clusters involved 4 patients (MS196, MS472, MS024 and MS351) with the hyper-toxin producing ribotype-027. Some clusters were epidemiologically linked in hospital at this visit (ribotype-027 group; MS196, MS472, MS024 and MS351) while other clusters had no hospital or community links in the last 90 days (ribotype-449 group; MS207, MS067 and MS363) determined using time and place analysis.

MMLVA identified 5 CD clusters with a total of 15 patients harboring highly related CD strains (Fig 4). Amongst the 5 clusters, one cluster of 2 individuals was colonized with a non-toxigenic strain of CD (ribotype-010), while a second cluster of 4 individuals was colonized with the highly virulent ribotype-027 strain (Fig 4). The other three clusters involved 2, 3 and 4 individuals colonized with ribotype-015, ribotype-449 and ribotype-081 respectively (Fig 4). While enrolled in the study, 4 patients included in the 5 patient cluster-groups developed CDI, 2 patients with ribotype-027 (MS196 and MS351) and the other two patients with ribotype-015 (MS200) and ribotype-081 (MS027) (Fig 4).

MMLVA and ribotyping analysis confirmed that 3/45 (6.7%) colonized patients (MS147, MS017 and MS424) harbored multiple CD strains (Fig 2; patients with *) including toxigenic and non-toxigenic isolates. In individuals with multiple strains, swabs were collected a median of 12 days apart (range 1–21 days). Individual MS017 was colonized with two toxigenic ribotypes (081 and a non-typed ribotype) and a non-toxigenic strain that was not ribotyped. The second individual, MS147, was colonized with two highly toxigenic CD strains (078 and 027) while the third asymptomatic carrier, MS424, was colonized with two un-typed toxigenic strains (Fig 2, S2 Data).

### Epidemiologic linkages

Epidemiologic investigations of two cluster, one with three individuals (MS207, MS067, and MS363) and the other consisting of four individual (MS408, MS414, MS027, and MS017) harboring identical CD strains (< 1% divergence) found that all of the patients within each cluster had overlapping hospital stays and patients were in adjoining rooms on the ward. In the case of one of the clusters, MS067 was positive on admission, while MS207 and MS363 were CD positive post admission, suggesting that transmission may have occurred between these patients. In contrast, the 4 individuals (MS196, MS472, MS024, MS351) with ribotype-027 strains that were identical by MMLVA were identified as colonized on admission to the hospital, and only one had been previously admitted to our hospital. The three other patients were transferred from 2 different long-term care facilities. Additional investigations of patient movements within the hospital and outside of the hospital based on their residential postal codes identified that 8/15 individuals in five clusters acquired their CD nosocomially at a time when other individuals colonized with the same strain were present on the same ward, while the remaining 7 individuals were colonized on admission to the hospital. Of the five clusters representative data is shown on three of the clusters (Fig 4). Of the four individuals identified as colonized with the ribotype-027 strain, two developed active CD disease within 90-days of study completion. As expected, the proportion of individuals that developed clinical CD disease was higher amongst ribotype-027 carriers compared to individuals carrying non-027 isolates (2/4 vs. 2/31 respectively, RR 7.7), although numbers for ribotype-027 carriage in this cohort are small.

## Discussion

Understanding the role asymptomatic carriers play on perpetuating CD transmission in healthcare settings is particularly challenging to examine, because most facilities do not screen for CD colonization. In patients with active CDI, contact precautions are used to manage and minimize CD transmission to other patients, however asymptomatically colonized individuals remain an unrecognized source of CD in the hospital environment[31]. A recent study found an asymptomatic CD carriage rate of 11.8% among hospitalized patients[32]. Here we present data demonstrating that 10% (45/474) of patients admitted to two general medicine wards over a three month period were asymptomatically colonized with CD, with half of them colonized on admission to the hospital. These figures likely underestimate the true rates of asymptomatic carriage as our swab based screening method is likely less sensitive than stool cultures, as was evident by the fact that we did not detect active CDI in five individuals[33]. Although studies have suggested that dry fecal swabs have comparable sensitivity to swabs dipped in stool for CD detection[34], these studies have all been performed in active CDI cases and not when assessing CD colonization, which would likely have a lower concentration of CD compared to active CDI cases. In addition, a significant proportion of participants in our study (37%) had only one swab collected and since CD shedding in the majority of asymptomatic carriers was picked up intermittently, we likely underestimate the proportion of asymptomatic carriage in our cohort. Intermittent detection of CD may be related to sampling variations as well as variable shedding patterns that may be a phenomenon of asymptomatic carriers of CD, something that should be factored-in if the prospect of screening asymptomatic carriers for CD is considered.

To determine how related the CD isolates from asymptomatic shedders were, we ribotyped the majority of CD isolates and then used MMLVA to distinguish between CD isolates of the same ribotype. Since MMLVA is sensitive to changes between strains and have been observed to change within the same CD isolates over time, we ensured that the changes observed were not related to instability in the MMLVA method but attributable to differences between CD strains. Stability of the MMLVA methodology was examined by testing multiple specimens collected over time from the same patient (Fig 3). In 34 patients with multiple swabs, MMLVA profiles were identical in 31/34 (91%) patients. In one patient, MS089, 15 swabs collected over a 67-day period yielded the same MMLVA type (< 1% difference in the 8/15 swabs evaluated; Fig 3). Changes in MMLVA profiles (> 1% between isolates) would have suggested either instability of the technique, in vivo evolution of CD isolates or the presence of multiple CD strains in one individual. In the 3/34 (9%) patients with different MMLVA types over the study period the three patients were colonized by multiple strains (Fig 3, MS017 and MS147).

Although, the specific role of asymptomatic carriers remains unclear, this study and others suggest that asymptomatic carriage plays a central role in the dissemination of CD in institutional settings. In a large multi-institutional study, *Eyre et al*. reported that in over a third of patients that acquired CD nosocomially and became carriers of CD had no exposures to patients with CDI in the community or hospital [35] suggesting that asymptomatic carriage plays a major role in CD dissemination. This, along with the detection of ribotype-027 in asymptomatic carriers suggests that colonized patients provide an important reservoir for disease and outbreak causing CD strains in institutional settings. It still remains unclear how much CD asymptomatic carriers shed and if this level of shedding is sufficient to support transmission and/or colonization of patients and the environment. Murine studies in asymptomatically colonized mice have demonstrated that exposure to clindamycin led to CD super-shedding phenotype that resulted in a 6-log increase in organisms and spore burden per gram of mice feces[36]. This CD super-shedding persisted for up to 4-weeks in immunocompetent mice[36] and was a strong predictor for the onset of severe CD disease in immunocompromised mice[36].

It remains unclear what factors are associated with asymptomatic carriage of CD, however in human[37] and murine[38] studies evaluating the impact of antimicrobials on CD colonization suggest that the risk of CD colonization following antimicrobial therapy may be related to intestinal concentration of antimicrobials, even when the antimicrobials harbor direct anti-CD activity. However, the development of asuper-shedder phenotype has not been studied in humans, as most studies do not enumerate amount of CD shed in stool. The development of a super-shedder phenotype in humans would have major implications on the role CD carriage plays in institutional transmission of CD and needs to be urgently evaluated. Carriage of toxigenic CD strains in animal studies was associated with a 10-fold increased risk of developing CD disease[39], independent of antimicrobial exposure or other risk factors[39].

The ribotype-027 strain was detected in four asymptomatically colonized patients on admission, three of four patients were admitted to our hospital from 2 different long term care facilities, and the fourth had multiple previous admissions to our hospital; thus, although these patients did not acquired the strain directly from each other, it is likely that their CD was acquired in a hospital or long term care facility from either a patient or the environment.

Ribotype analysis of CD isolates revealed that multiple CD strains were found in a small proportion of asymptomatic carriers (Fig 4). Carriage of multiple CD strains in one individual included both a toxigenic and non-toxigenic strain. The impact of this remains unclear as the presence of non-toxigenic strains has been associated with lower CDI rates[40]. However, horizontal transfer of the pathogenic locus from toxigenic strains to non-toxigenic strains resulting in both strains becoming toxin producers[41] has also been observed. Furthermore, isolating asymptomatic carriers of CD resulted in a marked reduction in nosocomial transmission in both hospital [42] and long-term care facility environments[43].

Our study has several limitations including the identification of a fairly limited number of asymptomatic CD carriers. The study likely underestimates the rates of CD carriage as rectal swabs would have significantly less organism burden compared to stool specimens as has been observed with VRE[44]. The study also used CD culture to initially select for CD followed by PCR testing, a method that is likely to be much less sensitive than direct testing of rectal swabs by PCR. Culture based screening also introduced additional biases in the analysis because CD colonies that did not morphologically look like CD would likely not have been chosen for PCR analysis.

In summary, both ribotyping and MMLVA analysis of CD stains colonizing hospitalized patients identified that asymptomatic carriers of CD can carry and shed multiple unrelated CD strains, including toxigenic and non-toxigenic strains (Fig 2). Although this study did not show transmission of CD strains from an asymptomatic carrier that then led to CD disease in another patient, we did observe identical CD strain in two asymptomatic carriers of CD with the same strain causing CD-disease in a third patient (Fig 4). These finding point to the fact that transmission of CD from asymptomatic patients is likely contributing to hospital outbreaks of CD and that screening for asymptomatic carriage of CD may be a valuable intervention during hospital outbreaks or in institutions with a high CD burden.

## Supporting information

S1 Data Database of recruited patients and swabs from asymptomatically colonized CD patients. (XLSX)

S2 Data Raw sequencing files for the molecular characterization of CD isolates. (ZIP)
