# Correction: Evidence of transmission of *Clostridium difficile* in asymptomatic patients following admission screening in a tertiary care hospital

**DOI:** 10.1371/journal.pone.0219579

**Published:** 2019-07-09

**Authors:** Prameet M. Sheth, Katya Douchant, Yvonne Uyanwune, Michael Larocque, Arravinth Anantharajah, Emily Borgundvaag, Lorraine Dales, Liz McCreight, Laura McNaught, Christine Moore, Kelsey Ragan, Allison McGeer, George Broukhanski

In Fig 1, the labels “CD PCR Negative” and “CD PCR Positive” are swapped. Please see the correct Fig 1 here.

**Fig 1:**
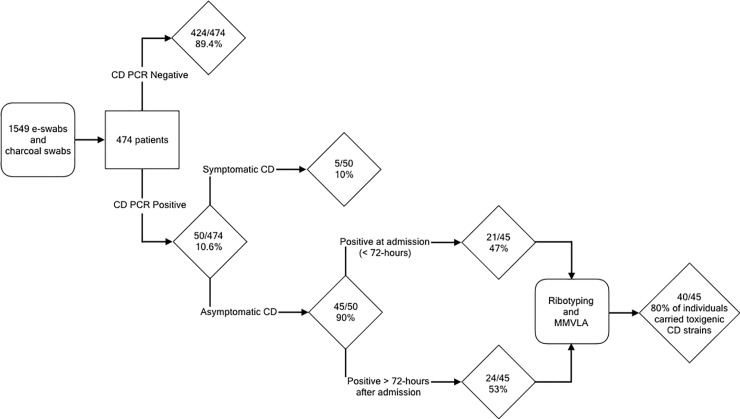
Schematic overview of enrolled study patients.
